# HOW CAREGIVERS DIVIDE RESPONSIBILITIES: A LATENT CLASS ANALYSIS OF US CAREGIVING ARRANGEMENTS

**DOI:** 10.1093/geroni/igad104.2596

**Published:** 2023-12-21

**Authors:** Erin Ice

**Affiliations:** University of Michigan, Ann Arbor, Michigan, United States

## Abstract

Family and other unpaid caregivers provide the bulk of caregiving for older adults, but we know little about how kin divide responsibilities amongst themselves, and how they share tasks with paid caregivers. This study identifies the common “care configurations” that emerge to support older adults with functional limitations. Studying older adults (age 65+) who received assistance from a caregiver in the National Health and Aging Trends Study Wave 5 (n = 4,182), I generate an extensive battery of caregiving arrangement indicators, including household, physical care, transportation, and administrative assistance provided by spouses, children, other unpaid kin, or paid caregivers. I use latent class analysis and adjust for survey design to identify common configurations. I find four primary care configurations. In the most common configuration (42%), partners provided all assistance and most often helped with household chores. Conversely, children often assisted with transportation while sharing other responsibilities with paid caregivers. Older adults with limited physical capacity, probable dementia, and Medicaid were most likely to belong to the only configuration featuring paid caregiving. By studying the full population of older adults who receive care assistance, this study provides a broad perspective on how older adults rely on unpaid kin caregivers and when they rely on paid caregiving. In particular, it identifies transportation assistance as an overlooked form of caregiving and demonstrates that children are most frequently dividing responsibilities with other kin or paid caregivers.

